# Tourism cooperation in the Belt and Road Initiative from economic and spatial insights

**DOI:** 10.1371/journal.pone.0300392

**Published:** 2024-05-20

**Authors:** Jie Yin, Yensen Ni, Yangchu Fan

**Affiliations:** 1 Department of Exhibition Economy and Management, Huaqiao University, Quanzhou, China; 2 Department of Management Sciences, Tamkang University, New Taipei, Taiwan; Universiti Malaysia Sabah, MALAYSIA

## Abstract

This study examines the potential benefits of cooperation among Belt and Road Initiative (BRI) countries in achieving common goals within the international tourism cooperation network. Despite its significance, limited research has been conducted on this topic in terms of economic and spatial insights. To address this gap, we utilized the gravity model, social network, and quadratic regression. The revealed findings suggest that while the intermediary function among BRI countries is declining, the tourism cooperation network is gradually strengthening. Furthermore, reducing the gap between the governance and consumption levels of BRI countries can improve the network. The study offers new insights into the BRI tourism cooperation network, which could be critical for the future growth of regional tourism.

## 1. Introduction

The absence of regional coordination and collaboration in tourism growth can lead to destination competition and slow economic development [1]. In contrast, cooperation between organizations and countries can result in advantageous partnerships that facilitate the attainment of shared objectives [2]. Previous research argued that regional tourism cooperation is the current trend of tourism development [3]. Consequently, numerous studies have explored the benefits of cooperation in an increasing range of fields, such as public administration [4] and tourism [5, 6]. Specifically, research in tourism cooperation has examined various aspects, including tourism attractions [7], tourism organizations [5], tourism destinations [8, 9], tourism enterprises [10], and tourism marketing [1]. Moreover, a country’s authority should improve transportation and facilities to enhance tourism cooperation with other countries [11]. In recent decades, cross-border tourism cooperation has also witnessed growth [12–16].

In the context of measuring tourism cooperation relationships, network theory posits that a network is composed of actors or "nodes," as well as the connections or "edges" between these actors [17, 18]. Actors within a network have various types of ties, including communication ties, formal ties, affective ties, material or workflow ties, proximity ties, and cognitive ties [17]. Network theory is commonly used to explain how countries interact, relate to one another, and evolve within a complex network [19]. However, most existing tourism cooperation studies focus on small-scale cooperation networks, such as destination stakeholder cooperation networks [5] and tourism enterprise cooperation networks [8, 10], with little attention paid to large-scale international tourism cooperation networks [15]. Therefore, we employ network theory to examine a large-scale international tourism cooperation network among BRI countries, which has been largely overlooked in current literature.

Furthermore, previous research has delved into the cooperation among countries across a range of sectors, including the economy and tourism [20]. Over the past few decades, tourism cooperation has become increasingly important due to its positive impact on the economy, including employment growth, increased income, and higher consumption in the tourism sector [21, 22]. However, as previous research has primarily focused on regional [23] and national cooperation [24], we contend that exploring tourism cooperation with BRI countries warrants further investigation. Many studies on tourism cooperation not only examine ways to enhance cooperation among BRI countries [25] but also explore tourism cooperation across various spatial scales.

We argue that this study may contribute to the existing literature. Firstly, unlike previous studies that focused on tourism enterprise cooperation networks, tourism research cooperation networks, and tourism stakeholders’ cooperation networks [26], we deconstruct a tourism cooperation network among BRI nations employing this gravity model and social network analysis. Second, in addition to uncovering various critical influential factors affecting such a network, we disclosed that narrowing the gap between BRI countries’ governance and consumption levels will enhance the network. Third, this network is steadily strengthening, and the function of "intermediary" among BRI countries is decreasing, signaling that authorities should direct the tourism cooperation network among BRI countries. We explain the roles of leader, intermediary, and autonomy among BRI countries and separate them into distinct blocks in the tourism cooperation network, including broker, sycophants, primary, and isolate blocks, both of which are underrepresented in the previous research.

The study aims to investigate two critical areas of concern. Firstly, it seeks to explore this network from 2000 to 2018 through a comprehensive analysis of global, small-group, and individual perspectives. Secondly, it intends to identify and examine the key driving factors of the tourism cooperation network in the BRI region. By addressing these two objectives, this study aims to contribute to the existing body of knowledge on BRI tourism cooperation and provide valuable insights for policymakers and practitioners in the tourism industry.

## 2. Research design

### 2.1. Tourism cooperation ties measurement

The gravity model, initially proposed in the 1960s, has continued to be widely applied for assessing network ties across various fields, including trade and urban development. Researchers have recently focused on studying economic relationships using the gravity model, and its effectiveness has been demonstrated in studies investigating economic links, making it a well-regarded model.

The proximity of economic distance to spatial distance [27–29] and the state of the industrial development environment [30, 31] are crucial determinants for tourists when making decisions about travel destinations. Consequently, in Eq (1), we have introduced modifications to the gravity model to gauge the level of tourism cooperation links, which take into account these two indispensable factors, namely the industrial development environment and economic distance.

$${TC}_{ij}=K_{ij}*\sqrt{{NT}_{i}*{TI}_{i}}*\frac{\sqrt{NT_{j}*TI_{j}}}{{GD}_{ij}*{ED}_{ij}},$$ (1)

where *TC*_*ij*_ denotes the ties of cooperation between nations i and j; *NT*_*i*_ and *NT*_*j*_ are the numbers of tourists in the nations *i* and *j*; *TI*_*i*_ and *TI*_*j*_ are the tourism income of nations *i* and *j*; *GD*_*ij*_ is determined by the distance between two national capitals; *K*_*ij*_ is the industrial development environment representing the tourism cooperation coefficient for countries *i* and *j* as shown in Eq (2); and *ED*_*ij*_ is determined by an Eq (3).

$$K_{ij}=\frac{SE_{i}}{SE_{i}+SE_{j}},$$ (2)

where *SE*_*i*_ and *SE*_*j*_ represent the proportion of service industry employment in total employment for countries *i* and *j*, which would be measured as the service industry’s industrial development environment.

Eq (3) utilizes the economic distance measuring technique to assess the economic distance.

$$ED_{ij}=\frac{{\left(GDPC_{i}-GDPC_{j}\right)}^{2}}{GDP_{i}*GDP_{j}},$$ (3)

where *ED*_*ij*_ denotes the economic distances between countries *i* and *j*; *GDPC*_*i*_ and *GDPC*_*j*_ denote the per capita GDP of countries *i* and *j*; and *GDP*_*i*_ and *GDP*_*j*_ denote the GDP of countries *i* and *j*.

### 2.2. Social network analysis

Given the ability of social network analysis to depict node relationships in a network, we employ this methodology to gauge both the collective and individual attributes of the network [7, 32]. Additionally, we employ the quadratic assignment procedure (QAP) approach [7], to further explore the principal factors driving the tourism cooperation network. Hence, we provide a succinct overview of the measurement of the network’s general, small group, and individual features, as well as the QAP analysis methodology, as follows.

1. Measuring the network’s overall characteristics: To measure the characteristics of the tourism cooperation network, several metrics can be used, such as network density, centralization, and E-I index [33]. Network density, which is the ratio of the actual number of links to the maximum number of possible links in the network [34], can provide insights into the connectivity of network entities. The higher the network density, the closer the entities are connected [35]. In addition, individual characteristics can be assessed through centralization measures such as degree centralization, betweenness centralization, and closeness centralization. These measures can indicate the degree of concentration within the network [35]. Another important metric is the E-I index, which is used to evaluate the level of the faction within the cooperation network. The E-I index has a possible range of -1 to 1, with a higher number indicating a larger degree of faction within the network and a lower value indicating a lesser degree of faction. The E-I index can also be negative. A figure that is close to zero may indicate that the faction’s position inside the corporate network is unclear [35].
2. Measuring the network’s small-group characteristics: An appropriate way for analyzing the features of small groups inside a network is to use the block model [36]. In order to assist understand the internal dynamics of small groups, Burt (1979) postulated four separate network positions, which included the isolate block, the sycophants block, the broker block, and the primary block [37]. An isolate block is characterized by limited interaction between members and the outside world, while a sycophants block features weaker internal ties than external ones. A broker block is defined by members who facilitate external relationships more than internal ones, while a primary block consists of members who primarily establish either external or internal relationships [37]. With the aim of evaluating small group cooperation within the BRI tourism cooperation network, we leverage the block model as a suitable analytical tool.
3. Measuring the network’s individual characteristics: In the evolution of a network, the structural hole, as measured by various indicators, plays a crucial role [38, 39]. Betweenness, effective size, efficiency, and constraints are common metrics used to measure the structural hole. Betweenness measures an organization’s ability to control information and act as an intermediary in a network. A higher value of betweenness indicates better control and mediation ability of members. Effective size is another indicator of control power, which measures the extent to which a node can influence others in the network. A higher effective size value suggests that the node has strong control power and is efficient in influencing other nodes [40]. Therefore, it is important to assess these metrics to understand the role of the structural hole in shaping the dynamics of a network.
4. The QAP regression: This study uses Quadratic Assignment Procedure (QAP) regression to analyze the causal link between the variables, which may effectively address the issue of multicollinearity [41]. The correlation coefficient is calculated using QAP regression. This aids in identifying the important aspects influencing the tourist collaboration network. This study can rapidly analyze the impact of several factors on the network topology using QAP regression while avoiding the issue of multicollinearity, which is a prevalent difficulty in regression analysis. As such, this technique may help us to get insights into the complex interactions between variables and their significance in shaping the BRI tourist cooperation network.

### 2.3. Selection of driving factors

Tourism activities may be significantly influenced by the distinction between the tourist source nation and the destination nation. As a result, we looked into the major determinants that affect the tourism cooperation network among BRI countries, concentrating on the differences across eight factors: security (SD), economics (ED), land adjacency (LA), tourism openness (TOD), population density (PDD), language (LD), consumption level (CLD), and governance (GD). These factors were chosen to develop a thorough knowledge of the motivations that drive international tourist cooperation while taking into consideration various ways that countries vary from one another.

1. Security difference (SD): When evaluating the driving factors affecting the tourism cooperation network among BRI countries, one crucial element is the differences in tourism security between the tourist source and destination countries. This factor has been shown to influence international tourism collaboration [16]. Intentional homicide rates are frequently used to gauge a country’s security situation. To assess the occurrence of a security gap between two BRI nations, we compute the average difference in intentional homicide rates over 19 years (2000–2018). This 19-year average difference serves as the baseline against which we measure each year. If the difference in intentional homicide rates between two BRI countries is less than the benchmark, we set SD as 0. This allows us to assess the impact of security differences on the tourism collaboration network among BRI countries.
2. Economic difference (ED): Differences in economic factors such as price, income, and currency rates can have a significant impact on tourist flow [42, 43]. As a proxy for determining the economic difference (ED) that exists between the two countries in the BRI network, we utilize the difference in gross national income (GNI). Specifically, we compare the GNI difference between the two countries with the benchmark, and if the difference is less than the benchmark, we set ED to 1. By using GNI as a proxy, we can effectively capture the economic situation and its potential influence on tourism activities in BRI countries.
3. Land adjacency (LA): Geographical distance, particularly land adjacency, is a critical factor in influencing tourism activities between countries. Studies have shown that neighboring countries have a significant impact on trade volume [44] and tourists’ destination choices [7]. Therefore, we use land adjacency as a proxy for the LA variable. If the two countries being compared are bordering countries, we assign LA a value of 1.
4. Tourist openness difference (TOD): Tourism openness (TO) is a crucial factor that influences tourism collaboration as it reflects the level of tourism development [45]. Cultural exchange and the positive image of a place can be promoted by tourism openness, leading to increased tourist inflow [46]. The measure of tourism openness is calculated as the sum of inbound and outbound tourism openness, weighted equally to represent the degree of tourism openness for a country. The inbound (outbound) tourism openness is computed as inbound (outbound) tourism revenue (spending) divided by the GDP of the country [47]. If the difference in tourism openness between two countries is less than the benchmark, we assign TOD a value of 0.
5. Population density difference (PDD): The population density of a country is an important determinant of tourism demand, as higher population densities are often associated with a greater propensity for overseas travel. Thus, we include population density as a driving factor in our analysis of tourism cooperation among BRI countries. Specifically, we calculate the population density difference between two distinct nations, and if this difference is smaller than the benchmark, we assign a value of 0 to PDD. Previous research has highlighted the value of population density as a predictor of tourism demand [48, 49], emphasizing its importance as a factor in our investigation of the BRI tourism network.
6. Language difference (LD): From a cross-cultural perspective, differences can play a significant role in boosting tourism activities [50, 51]. Among these differences, language is considered a crucial factor since it has a significant impact on international tourism activities and enhances the tourism experience [50, 52]. Tourists tend to travel to countries where they can communicate in the same language [53]. Thus, we consider the language difference as a crucial driving factor affecting tourism cooperation. Specifically, if two distinct BRI countries share the same official language, we set LD to 1.
7. Consumption level difference (CLD): The level of consumption in a destination may reflect the cost of a trip to that destination. Given that tourism activities are significantly impacted by the cost of traveling, Timothy and Kim (2015) argued that travel expenses are one of the most important factors influencing tourism demand [16]. To assess the level of consumption, we utilize per capita consumption. If the difference in per capita consumption between two distinct BRI countries is less than the benchmark, we set CLD to 0.
8. Governance difference (GD): To enhance tourism development, a country must have robust governance structures in place [54, 55]. Therefore, governance difference (GD) is a crucial factor to consider in tourism promotion [56]. To measure GD, the World Bank employs six indicators including voice and accountability, political stability and absence of violence/terrorism, government effectiveness, regulatory quality, rule of law, and control of corruption. Prior research suggested averaging these six indicators to assess the governance ability of BRI countries [57]. If the difference between the governance abilities of two BRI countries is lower than the benchmark, GD is set to 0.

### 2.4. Data sources

The primary objective of tourism cooperation among BRI members is to increase tourist inflows, thereby generating higher tourism income for these countries. To achieve this objective, we use international tourist arrivals and international tourism income as important indicators rather than total tourist arrivals and tourism income in a country, as Eq (1) demonstrates.

To assess the industrial development environment, this study uses service employment as a percentage of total employment [47]. The data for this analysis was obtained from the World Bank’s World Development Indicators database. Additionally, the CEPII database was used to calculate the spatial distance between two capitals as a measure of the geographic distance between two BRI countries. A total of 60 BRI countries were included in this analysis over the 2000–2018 period, as data was unavailable for six countries, namely Iraq, Syria, Afghanistan, Turkmenistan, and Uzbekistan. Due to the difficulty in creating country-pairs data, these 60 BRI countries were either considered as source or destination countries in this study.

In terms of the driving factors, intentional homicide rates were collected from the United Nations Office on Drugs and Crime’s International Homicide Statistics database (http://www.unodc.org/) to assess a country’s security. The World Bank provided data on inbound tourism revenue, outbound tourism spending, GDP, population density, per capita consumption, gross national income (GNI), and governance including voice and accountability, political stability and absence of violence/terrorism, government effectiveness, regulatory quality, rule of law, and control of corruption. Additionally, data on the official languages of BRI countries was sourced from the French CEPII database. The study collected data from 2000 to 2018 for this analysis.

## 3. The international tourism cooperation’s characteristics

### 3.1. The network’s overall characteristics

**Fig 1:**
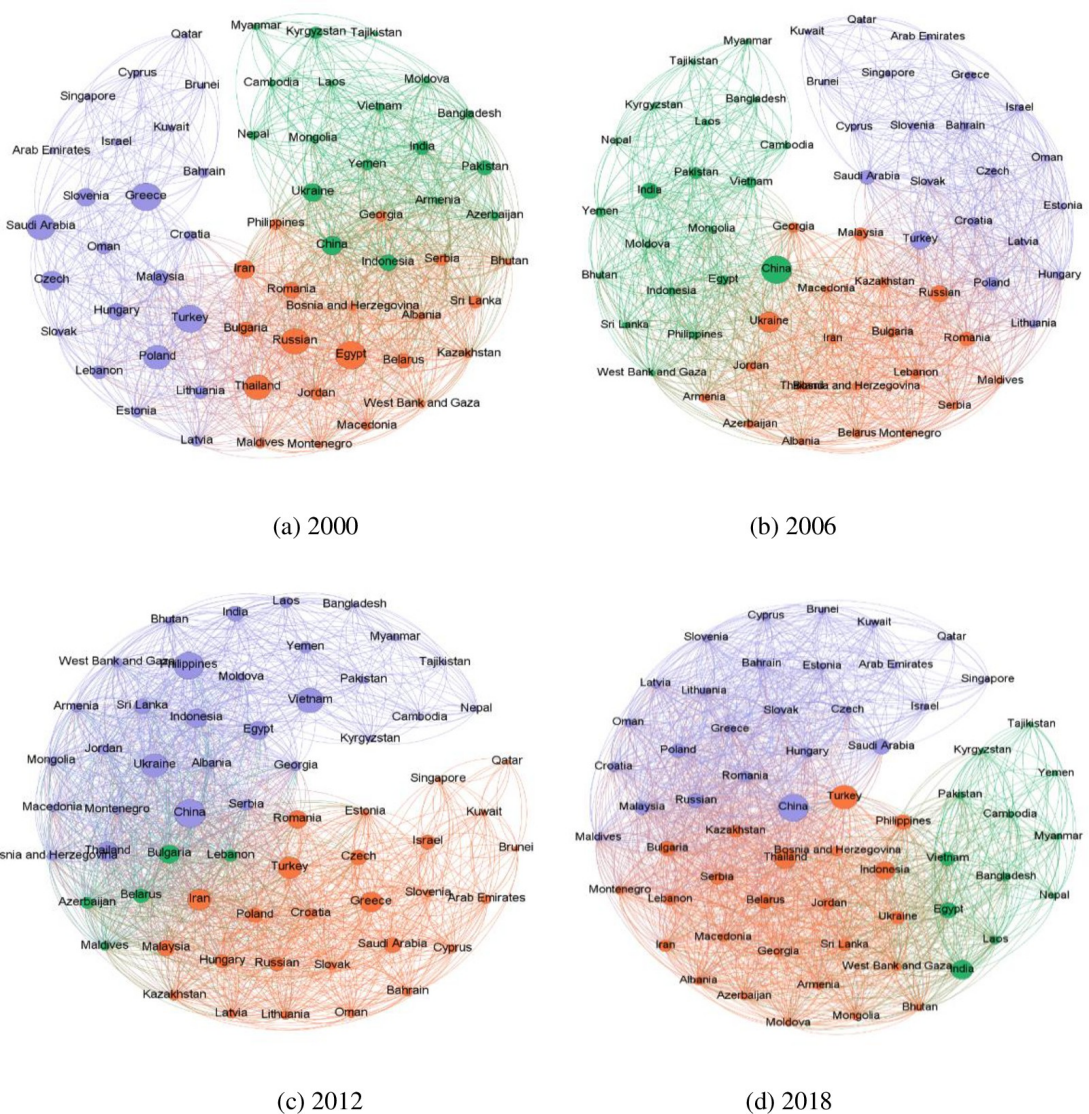
Tourism cooperation networks among BRI countries in 2000, 2006, 2012, and 2018. (a) 2000, (b) 2006, (c) 2012 and (d) 2018.

We have constructed a tourism cooperation relationship matrix using the revised gravity model and have visually represented the tourism cooperation network for four stages, specifically in 2000, 2006, 2012, and 2018, as illustrated below (Fig 1) by employing the Ucinet and Netdraw software. The average value of tourism collaboration ties from 2000 to 2018 was utilized as the breakpoint value in this analysis so that we could assess the value of the relationships between the tourism organizations. If the value between two BRI nations is greater than the mean, this study sets the value of the relationship to 1, and if it was lower than the average value, we set it to 0. It can be seen that the network of tourism collaboration is getting increasingly intertwined as the number of cooperation relations between BRI countries increased from 1308 in the year 2000 to 1423 in the year 2006, 1693 in the year 2012, and 1737 in the year 2018. The steady increase in the number of cooperation ties over the years suggests the potential for further growth and collaboration within the tourism industry.

**Fig 2:**
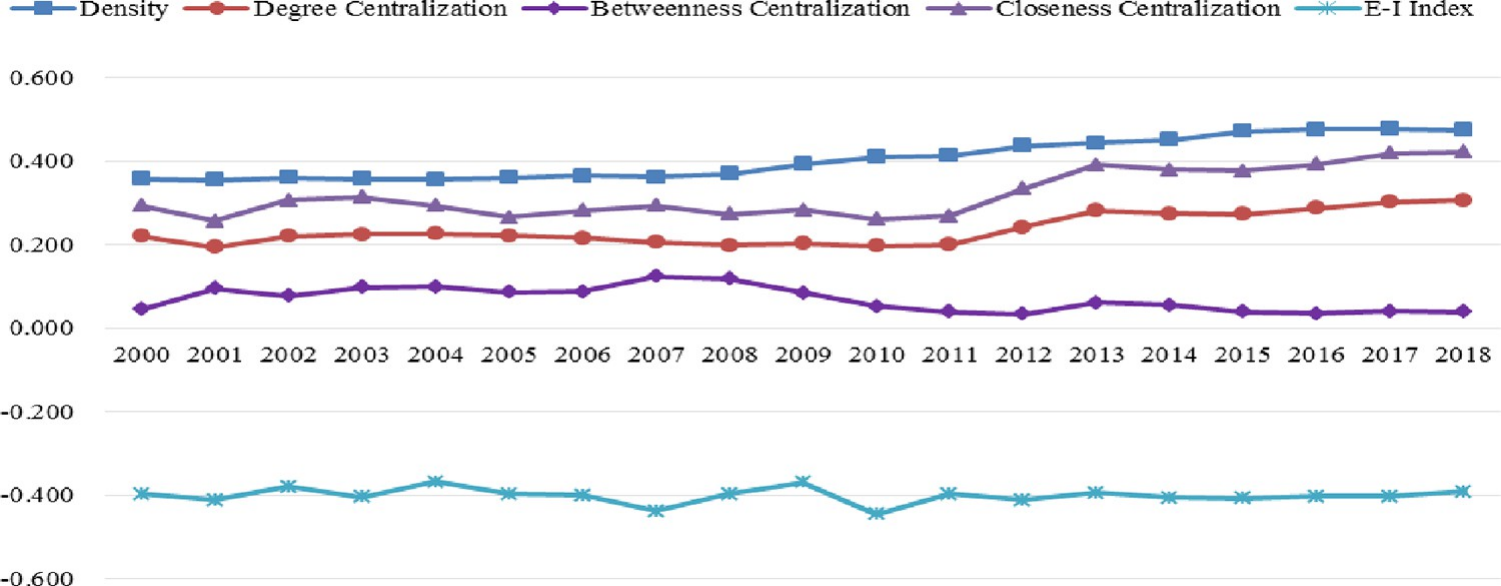
The overall characteristics of the tourism cooperation network from 2000 to 2018.

In addition, this study employs network density, E-I index, and centralization to evaluate the comprehensive properties of the tourism collaboration network across the BRI. The outcomes of this analysis are illustrated in Fig 2.

Regarding network density, our findings reveal a gradual increase from 0.3574 in 2000 to 0.4746 in 2018, indicating an increment in the extent of interconnectivity among the participating countries. The centralization of the network displayed a slow increase over time but remained below 40%, indicating the absence of any dominant player in the network. However, the betweenness centralization declined, suggesting that the role of intermediaries has diminished due to increased direct collaboration among the member states. This is a noteworthy finding that could impact future tourism-related policies and cooperation. Additionally, the closeness centralization showed a fluctuation of around 30% higher than other characteristics, indicating that the links between "central countries" and "edge countries" (In this study, central countries are defined as those that may have a strong economy and consumption power, whereas edge countries are those that may not have a strong economy and consumption power) need strengthening. Furthermore, the E-I index exhibited a narrow range of fluctuation around -0.4, indicating the possibility of fractional relations among the BRI countries. We detected patterns of small group cooperation in the network, highlighting opportunities to enhance overall tourism collaboration in the future.

### 3.2. The network’s small group characteristics

The E-I index has revealed that the tourism cooperation network consists of small groups. To explore this further, we have utilized the Convergence of Iterated Correlations (CONCOR) method to cluster the BRI countries, with a maximum segmentation of 2 and a convergence criterion of 0.2. Through this method, we have successfully partitioned the selected 60 BRI countries into four distinct blocks [40]. Before discussing our findings, we will first elucidate the roles played by each of these blocks.

#### 3.2.1. The role of various blocks

**Fig 3:**
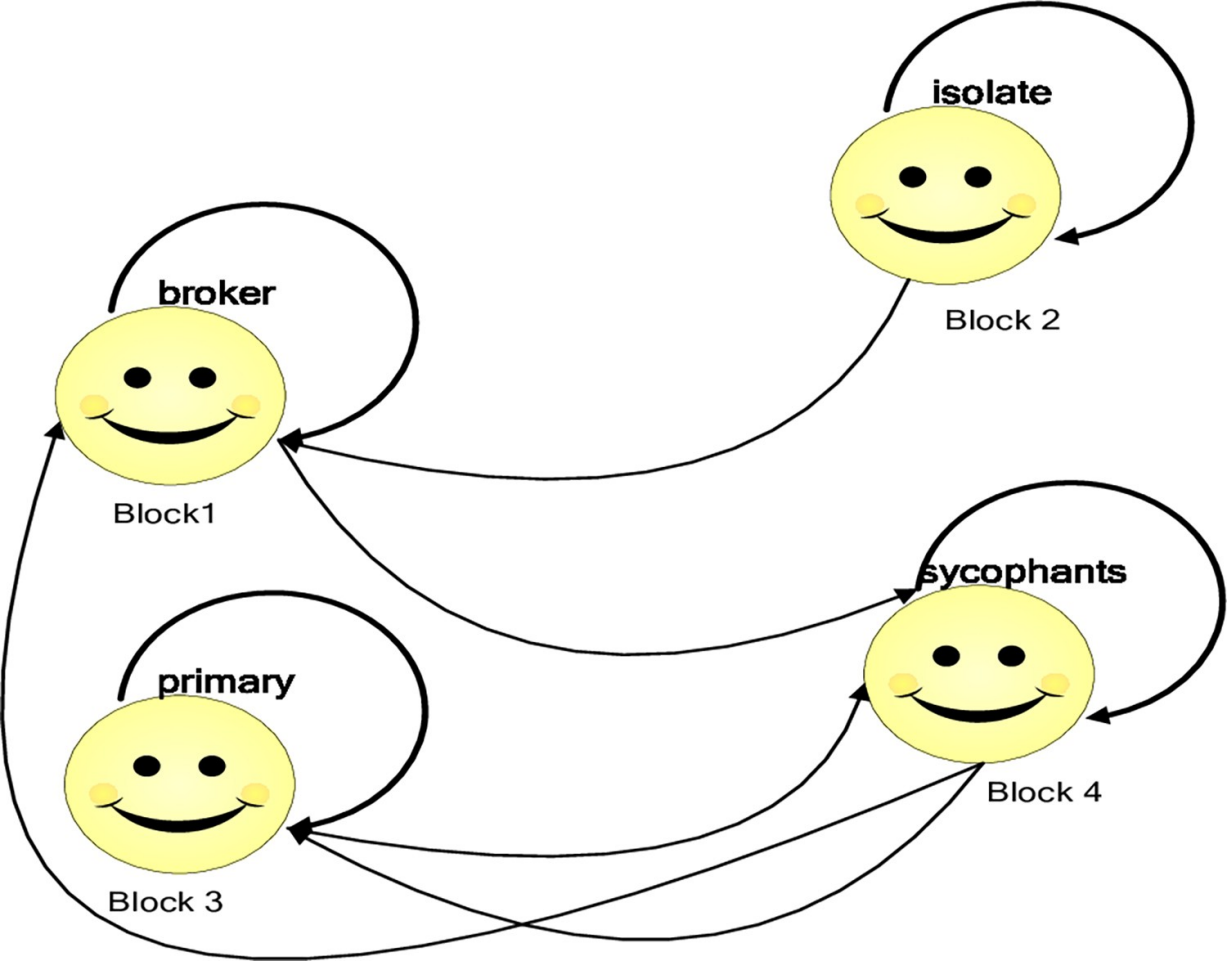
The role of various blocks in the tourism cooperation network.

Based on the image matrix for each year, this study has developed a succinct relationship diagram depicting the roles of various blocks, namely block 1, block 2, block 3, and block 4, as presented in Fig 3. The arrow situated above each block represents the relationship "sent" by a block, which "returns" to this block (Some members in a block contact with other members in the block each other, indicating some members "send" messages to other members, and other members "return" messages to some members after receiving the message), suggesting that each block has specific relationships with internal members.

Fig 3 reveals that block 1 not only possesses relationships among internal members but also accepts the relationships initiated by block 2 and sends the relationship to block 4. This observation indicates that block 1 may function as a "bridge" to facilitate communication between block 2 and block 4, rendering it the broker block. Conversely, block 4 may be considered the sycophant block since it frequently receives and sends relations for other blocks, including block 1 and block 3, instead of its own.

Concerning block 3, it can be discerned that it serves as the primary block because it not only sends and receives relationships from block 4 but also has a close relationship with its internal members. Moreover, block 2 could be considered the isolate block since, compared to other blocks, it seldom receives and sends relationships to other blocks.

#### 3.2.2. The members of the various blocks

**Fig 4:**
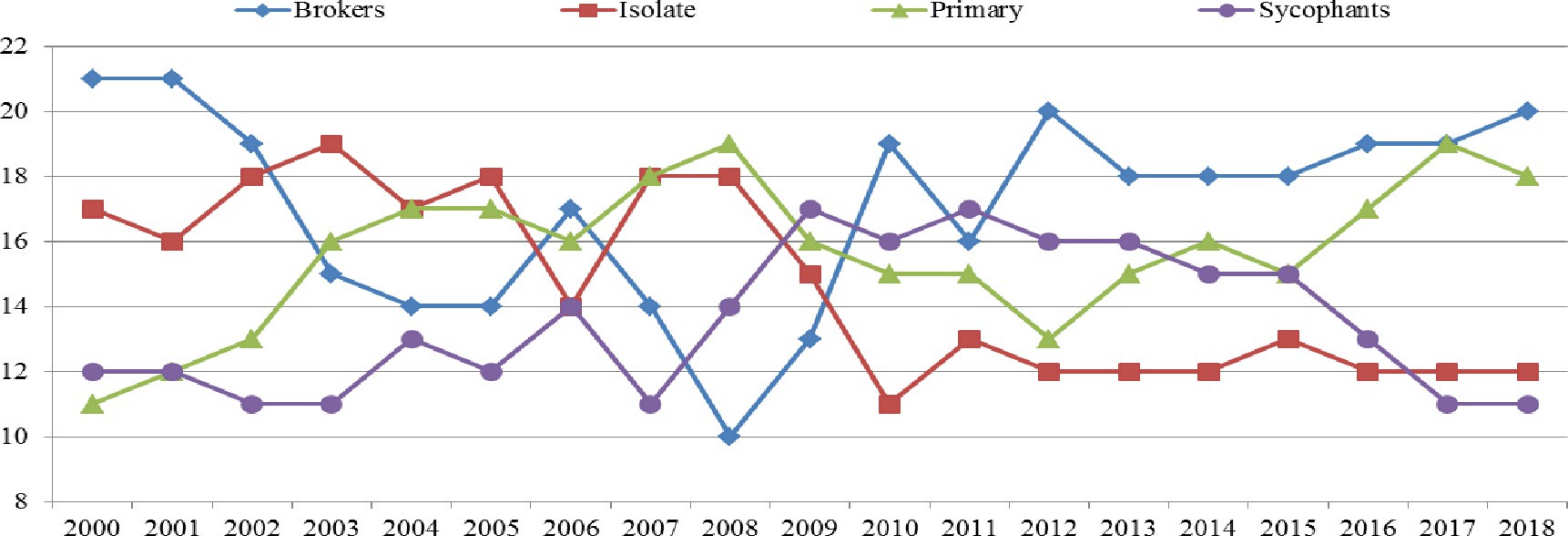
The number of BRI countries changed in four distinct blocks from 2000 to 2018.

In this study, we employed Ucinet software to calculate the density between blocks, assigning a value of 1 if the density was greater than 1 and 0 otherwise. Using this information, we constructed an image matrix to analyze the relationship between blocks and divided BRI countries into various blocks. As demonstrated in Fig 4, the members in these blocks vary across the 2000–2018 timeframe.

As depicted in Fig 4, the number of members in the isolate blocks gradually decreased over time, indicating that the isolate role may no longer be suitable for current cooperation mainstreams. On the other hand, the number of members in the prime block has shown an upward trend, suggesting that there has been an improvement in tourism cooperation among BRI countries. However, the overall number of brokers has decreased, indicating that intermediaries may no longer play as critical a role in tourism cooperation as they once did.

In addition, it can be observed from Fig 4 that the members in the sycophants block exhibit a rising trend from 2000 to 2009, followed by two decreasing trends after 2009, indicating a higher level of volatility compared to other blocks. It is noteworthy that by the end of the data period, the number of "outward-oriented" members in the sycophants block, i.e., those with a tendency to receive and send relationships for other blocks, dropped to less than ten nations for tourism cooperation.

Overall, this study suggests that the interaction among BRI countries, including direct cooperation, has been improving from 2000 to 2018, as evidenced by the upward trend of the primary block, which contains more countries compared to other blocks, as shown in Fig 4. In this study, 60 BRI nations were categorized into different blocks for the tourism cooperation network in 2018, including the broker block, isolate block, primary block, and sycophants block, as illustrated in Table 1.

**Table 1:** The Block type of 60 countries in 2018.

Block type	Countries
Broker block	20 countries: Albania, Georgia, Mongolia, Macedonia, Azerbaijan, Moldova, Armenia, Serbia, Belarus, Iran, Ukraine, Indonesia, Bosnia, Bhutan, Thailand, Philippines, Jordan, Lebanon, Sri Lanka, Palestine
Isolate block	12 countries: Egypt, Bangladesh, Kyrgyzstan, Laos, Cambodia, Yemen, Vietnam, India, Myanmar, Pakistan, Nepal, Tajikistan
Primary block	18 countries: Saudi Arabia, Israel, Greece, United Arab Emirates, Bahrain, Brunei, Cyprus, Kuwait, Czech Republic, Slovakia, Estonia, Oman, Lithuania, Qatar, Singapore, Hungary, Latvia, Slovenia
Sycophants block	10 countries: Bulgaria, Kazakhstan, Turkey, Malaysia, Maldives, Poland, Croatia, Russia, Romania, China

### 3.3. The network’s individual characteristics

This study has evaluated the individual characteristics of the BRI countries, such as effective scale, efficiency, constraints, and centrality among these countries. The findings are presented in Table 2.

**Table 2:** Ranked first and last based on various individual attributes over the period 2000–2018.

year	Effective scale		Efficiency		Constraints		Betweenness	
	nation	measure	nation	measure	nation	measure	nation	measure
2000	Russia	15.902	Russia	0.442	Tajikistan	0.192	Russia	8.865
	Tajikistan	2.132	Tajikistan	0.152	Russia	0.094	Tajikistan	0
2001	Russia	17.161	Russia	0.477	Myanmar	0.188	Russia	9.212
	Israel	2.817	Israel	0.156	Russia	0.091	Israel	0
2002	Russia	16.057	Russia	0.459	Myanmar	0.214	Russia	10.85
	Myanmar	3.205	Brunei	0.166	Russia	0.093	Israel	0
2003	Russia	16.75	Russia	0.465	Tajikistan	0.163	Russia	12.026
	Greece	3.372	Greece	0.161	Russia	0.094	Laos	0
2004	China	15.898	China	0.454	Myanmar	0.169	Jordan	9.931
	Greece	3.025	Greece	0.144	Jordan	0.097	Myanmar/Greece	0
2005	Jordan	17.392	Jordan	0.47	Myanmar	0.177	Jordan	9.931
	Myanmar	3.3	Myanmar	0.145	Jordan	0.095	Bahrain	0
2006	Ukraine	16.746	Ukraine	0.453	Myanmar	0.175	Ukraine	6.82
	UAE	2.967	UAE	0.141	Jordan	0.094	UAE/Myanmar	0
2007	Jordan	17.068	Jordan	0.461	Myanmar	0.173	Jordan	7.225
	UAE	2.691	UAE	0.122	Lebanon/Ukraine	0.093	UAE/Myanmar	0
2008	Jordan	16.608	Jordan	0.449	Bangladesh	0.173	Jordan	5.94
	Bangladesh	3.242	UAE	0.138	Ukraine	0.091	UAE/Myanmar	0
2009	Jordan	19.47	Jordan	0.475	Bangladesh	0.158	Jordan	6.676
	Greece	3.533	Greece	0.141	Jordan/China	0.085	Bangladesh/Greece	0
2010	China	20.865	China	0.454	Bangladesh	0.151	China	5.887
	Cyprus	4.177	Cyprus	0.155	Thailand	0.079	Bangladesh	0
2011	China	22.203	China	0.463	Bangladesh	0.16	China	7.835
	Bangladesh	3.532	Brunei	0.139	China	0.076	Bangladesh	0
2012	China	24.924	China	0.462	Bangladesh	0.156	China	7.896
	Brunei	3.289	Brunei	0.132	China	0.074	Bangladesh	0
2013	Russia	15.902	Russia	0.442	Tajikistan	0.192	Russia	8.865
	Tajikistan	2.132	Tajikistan	0.152	Russia	0.094	Tajikistan	0
2014	China	23.603	China	0.445	Pakistan	0.139	China	5.088
	Kuwait	2.722	Kuwait	0.118	China	0.073	Kuwait	0
2015	Thailand	23.583	Thailand	0.437	Cambodia	0.127	Thailand	4.538
	UAE	3.561	Kuwait/UAE	0.127	Thailand	0.073	Yemen	0
2016	China	22.42	China	0.423	Yemen	0.133	Thailand	3.052
	UAE	3.429	UAE	0.122	China	0.073	Yemen	0
2017	China	18.052	China	0.376	Qatar	0.401	China	5.199
	Tajikistan	1.083	Tajikistan	0.108	China	0.084	Tajikistan	0
2018	China	18.326	China	0.382	Qatar	0.402	China	5.105
	Tajikistan/ Yemen	1.167	Tajikistan/ Yemen	0.117	China	0.084	Tajikistan/ Yemen	0

Note: United Arab Emirates is abbreviated as UAE

Our analysis reveals a decline in the role of "intermediaries" for BRI countries over the years, as shown by the decrease in the maximum centrality value from 8.865 (Russia) in 2000 to 5.105 (China) in 2018. This trend suggests that direct cooperation among BRI countries is gaining prominence, reducing the need for intermediaries.

We also observe a trend of increased cooperation and intensity among the BRI countries, as indicated by the rising effective scale values in Table 2, irrespective of a country’s minimum and maximum scale. China, in particular, has consistently held the top rank in the effective scale since 2010, signifying its crucial role in the BRI tourism cooperation network.

In terms of efficiency, our analysis demonstrates a changing trend in the most dominant country, with Russia, Jordan, Ukraine, Thailand, and China taking on this role at different times. However, the overall efficiency of the BRI network is declining, implying a shift towards equality and balance among the participating countries.

Finally, our analysis indicates a gradual decrease in constraints for the BRI countries, reflecting the improvement in cooperation liberalization and facilitation. We suggest that countries with lower levels of cooperation liberalization, such as Tajikistan, Myanmar, Bangladesh, Pakistan, Cambodia, and Yemen, should increase their cooperation with other countries to further enhance their tourism networks.

## 4. The BRI tourism cooperation network’s driving factors

Utilizing QAP regression, this study examines the potential influences of security difference (SD), economic difference (ED), land adjacency (LA), tourism openness difference (TOD), population density difference (PDD), language difference (LD), consumption level difference (CLD), and governance difference (GD) on the tourism cooperation network among BRI countries. The findings, as shown in Table 3, shed light on the impact of those factors on tourism cooperation.

**Table 3:** The QAP regression analysis.

Year	SD	ED	LA	TOD	PDD	LD	CLD	GD	R^2^		Adj-R^2^
2000	-0.005	**0.050** ***	**0.041** ***	0.006	0.019	0.012	**-0.521** ***	**-0.131** ***		0.336	0.335
2001	0.001	**0.046** ***	**0.027** *	-0.007	0.021	0.015	**-0.524** ***	**-0.134** ***		0.344	0.343
2002	0.001	**0.028** *	**0.034** **	-0.017	0.021	**0.022** **	**-0.539** ***	**-0.122** ***		0.355	0.354
2003	-0.009	**0.030** *	**0.029** **	-0.032*	0.020	**0.019** **	**-0.537** ***	**-0.138** ***		0.365	0.364
2004	0.002	**0.026** *	**0.023** *	-0.034*	0.015	**0.024** **	**-0.535** ***	**-0.133** ***		0.363	0.362
2005	-0.004	**0.034** **	0.020	-0.020	-0.013	**0.012** **	**-0.532** ***	**-0.149** ***		0.363	0.362
2006	0.001	**0.040** **	0.015	-0.014	-0.002	**0.012** **	**-0.532** ***	**-0.119** ***		0.345	0.343
2007	0.009	**0.032** *	0.002	-0.014	-0.012	**0.008** **	**-0.525** ***	**-0.134** ***		0.344	0.343
2008	-0.018	0.021	0.005	0.002	-0.002	0.004	**-0.545** ***	**-0.131** ***		0.364	0.363
2009	-0.002	**0.032** **	-0.016	-0.017	-0.023	0.005	**-0.565** ***	**-0.150** ***		0.401	0.400
2010	**-0.034** ***	0.026	-0.003	0.000	-0.014	-0.010	**-0.564** ***	**-0.136** ***		0.391	0.390
2011	**-0.031** ***	0.021	-0.009	-0.005	-0.014	-0.010	**-0.547** ***	**-0.139** ***		0.368	0.367
2012	**-0.012** **	**0.038** *	-0.001	-0.002	-0.001	-0.008	**-0.559** ***	**-0.129** ***		0.363	0.362
2013	**-0.018** **	**0.032** *	-0.008	0.011	0.006	0.000	**-0.583** ***	**-0.126** ***		0.389	0.388
2014	-0.010	**0.038** **	0.004	-0.014	0.002	0.005	**-0.584** ***	**-0.115** ***		0.388	0.387
2015	0.003	**0.039** **	0.000	0.007	0.016	0.003	**-0.640** ***	**-0.110** ***		0.456	0.455
2016	-0.011	**0.063** ***	-0.009	-0.002	0.004	0.004	**-0.642** ***	**-0.122** ***		0.469	0.468
2017	-0.002	**0.067** ***	-0.019	0.036	0.005	0.003	**-0.647** ***	**-0.103** ***		0.468	0.467
2018	0.009	**0.041** ***	-0.004	0.006	**-0.049** **	-0.004	**-0.690** ***	**-0.111** ***		0.540	0.539

*** Statistically significant at 0.01 level

** Statistically significant at 0.05 level

*Statistically significant at 0.10 level

We find that, except for the 2010–2013 period, security differences do not significantly affect tourism cooperation. This result could be attributed to increased attention to tourist security among countries. Thus, other factors may play a more significant role in promoting tourism cooperation. Conversely, economic differences demonstrate a positive and robust impact on tourism cooperation. This finding suggests that countries with varying national incomes are more likely to cooperate in tourism, as tourists may prefer to visit countries with diverse infrastructure, pricing, consumption levels, and other factors.

Our analysis reveals that land adjacency has a favorable impact on tourism cooperation from 2000 to 2004, indicating that boundary tours continue to dominate tourism cooperation. However, the effect of land adjacency on tourism cooperation is gradually decreasing, as seen in the falling coefficients from 2005 to 2018. Thus, tourism cooperation may eventually break through regional cooperation and expand to global cooperation.

Tourism openness, defined as the percentage of incoming/outgoing tourism revenue and spending to GDP, is negatively and significantly related to tourism cooperation only in 2003 and 2004, suggesting that tourism openness differences may not weaken tourism cooperation after 2004. Population density differences have a complex influence on tourism cooperation, as we observe favorable impacts from 2000 to 2004 and from 2013 to 2017, but detrimental effects from 2005 to 2012 and 2018. This finding implies that population density differences may hinder tourism activity.

Language differences have a favorable impact on tourism cooperation, with positive effects observed from 2002 to 2007 and throughout the research period. This finding indicates that differences in traditional culture, humanistic environment, and resource endowment may attract tourists and promote tourism cooperation. In contrast, differences in consumption levels tend to impair tourism collaboration, suggesting that tourism cooperation may not be strengthened if countries have vastly different consumption levels. This finding could be attributed to tourists’ quality concerns and budget constraints.

Finally, we find that governance differences are consistently negatively related to tourism cooperation year after year, highlighting governance as a significant impediment to tourism cooperation. The development of tourism is aided by strong governance, but considerable governance differences between countries could severely hinder tourism cooperation. Therefore, we suggest that BRI nations’ governments should strive to improve their governance to enhance tourism cooperation.

## 5. Concluding remarks

### 5.1. Conclusion and discussion

The study aimed to deconstruct the tourism cooperation network among Belt and Road Initiative (BRI) countries by employing the modified gravity model and social network analysis to analyze the network’s characteristics and driving factors. Our results provide a comprehensive overview of the tourism cooperation network among BRI countries and reveal several crucial findings.

First, we find that the tourism cooperation network among BRI countries is becoming more closely connected, which may lead to a decrease in the importance of leadership and brokers within the network. The network’s increasing autonomy and the existence of small-group cooperation suggest that no single entity can dominate the network. This close tourism cooperation can be attributed to the growth of international tourism cooperation and frequent international tourist flows, resulting in greater tourism collaboration among BRI countries. To some extent, the close tourism cooperation among BRI countries may be attributed to growing international tourism cooperation [58] as well as frequent international tourist flows [19, 59]. That is, the closer tourism cooperation among BRI countries may result from the rapid growth of international tourism [52].

Second, our analysis indicates that the tourism cooperation network consists of brokers, sycophants, primary, and isolated blocks, and small group cooperation exists within the network. Our findings demonstrate that isolated block members are decreasing while primary block members are generally increasing, indicating that the isolation function is not suitable for current tourism collaboration among BRI countries. Additionally, Central Asia and Islamic countries have been gaining increasing attention in tourism cooperation. A cooperative relationship is a viable alternative, whereas isolation may be impractical [10]. Recently, in addition to BRI tourism cooperation, regional cooperation such as China-ASEAN [60], Central Asia [61], and Islamic nations [62] has received much more attention.

Third, we find that cooperation limitations among BRI nations are gradually decreasing, and the trend toward liberalized cooperation is becoming more apparent. The betweenness of intermediaries has weakened, indicating that direct linking between BRI countries would be more visible. Positive collaboration effects may make direct cooperation popular [63, 64], leading to the reduction of cooperation constraints or the role of intermediaries. As the restraints on BRI countries are steadily eliminated, the tourism cooperation network will become more liberated, and some countries with major influence will shrink, leading to greater equality within the network. China plays a significant role in the tourism cooperation network, as indicated by its top ranking in efficiency and its position as the world’s largest provider of outbound tourism and a major recipient of tourists. Since 2012, China has become the world’s largest source of outbound tourism [65] as well as one of the world’s primary recipients of tourists [66], since the number of inbound visitors has increased dramatically.

Finally, our QAP regression analysis identifies differences in security, tourism openness, population density, consumption level, and governance as negative factors affecting the tourism cooperation network among BRI countries, while differences in economics, language, and land adjacency have positive effects, which is consistent with previous research [50, 52, 67].

In conclusion, our study provides a comprehensive deconstruction of the tourism cooperation network among BRI countries, offering insights into the characteristics and driving factors of the network. Our findings suggest that increased direct linking, reduced cooperation constraints, and greater equality within the network will facilitate more extensive and effective tourism collaboration among BRI countries.

### 5.2. Research implications

Based on the study’s findings, we found several implications that could have a substantial impact on the tourism industry in BRI countries. First, the research recommends that BRI countries focus on enhancing their tourism marketing and management. Given that tourism is less harmful and provides limitless business prospects, it represents a fantastic chance for these countries to enhance their economies and living standards. However, to attract tourists, it is critical to improve not only marketing activities, but also tourism infrastructure management, such as roads, rest spots, and traffic. This can be accomplished by increasing the number of management employees and giving tourists clear recommendations for navigating the destination.

Second, the use of social media platforms like Facebook, YouTube, and Twitter to promote tourist destinations and make a favorable impression on tourists is an appealing option. In addition, word-of-mouth advertising is an efficient method for promoting a company that also can bring in a greater number of customers. As a result, the tourism industry can stand to gain from this form of advertising if it is done well.

Third, because the association between poverty and social unrest may not be low, this study anticipates that the economy of those countries will improve, resulting in a drop in those living in poverty and an increase in those able to have a better living standard. Thus, the tourism industry can contribute to the overall well-being of these countries by providing economic opportunities for the local population.

However, it is important to note that the severe impact of COVID-19 may have long-lasting effects on the tourism industry and the benefits outlined above may be difficult to demonstrate in the short term. Despite this, we believe that the findings of this study can help guide future efforts to promote and develop the tourism industry in BRI countries.

### 5.3. Limitations and future research

Despite uncovering the key characteristics and driving sources of the network, this study is not without limitations. First, while we employed a range of methodologies such as the revised gravity model and social network analysis, which have been supported by previous studies, we must still justify why our methods are superior in deconstructing the tourism cooperation network. Thus, further research is necessary to examine this issue thoroughly. Second, language differences serving as a proxy for culture, which is crucial factor influencing tourism cooperation, may be considered a limitation of this study. In future research, it may be worthwhile to explore alternative proxies for the culture that could provide more accurate assessments. Third, given the establishment of regional cooperation organizations, our study identifies the existence of small cooperative groups within the tourism cooperation network. Further investigation into the essential cooperative characteristics of these small groups and the mechanisms of tourism cooperation formation among them could provide valuable insights for future research.

## Supporting information

S1 Data (ZIP)
